# Bladder Cancer Cells Maintain Paracrine IL-1 Signaling and IL-1Ra Sensitivity Following Chronic IL-1 Exposure

**DOI:** 10.3390/cells15151376

**Published:** 2026-07-30

**Authors:** Jessica Gomez, Meron Lakew, Haley Wilkie, Bernice David, Oluwatamilore Taiwo, Roopal Dhar, Anusha Akula, Akshaykumar Thasma, Jeffrey Cho, Neil Sharma, Obinna Okafor, Nikki A. Delk

**Affiliations:** Department of Biological Sciences, The University of Texas at Dallas, Richardson, TX 75080, USA; jeg190011@utdallas.edu (J.G.); meronlakew100@gmail.com (M.L.); hcd160130@utdallas.edu (H.W.); bernice.david12@gmail.com (B.D.); taiwotamilore@gmail.com (O.T.); roopal.dhar@utdallas.edu (R.D.); anushaakula1106@gmail.com (A.A.); akshaykumar.thasma@utsouthwestern.edu (A.T.); jeffcho224@gmail.com (J.C.); n.sharma7570@gmail.com (N.S.); obinn.okafor@gmail.com (O.O.)

**Keywords:** interleukin-1, chronic inflammation, autocrine signaling, paracrine signal, bladder cancer, endothelial cell activation

## Abstract

Background: Cancer cells live in a dynamic and favorable ecosystem conducive to their growth and survival, known as the tumor microenvironment (TME). The TME is replete with various proinflammatory cell types that mediate crosstalk via the secretion of cytokines and chemokines to facilitate tumor growth and development. One cardinal proinflammatory cytokine that is present in the TME is interleukin-1 (IL-1). IL-1 promotes tumor angiogenesis and cancer cell metastasis; thus IL-1 receptor antagonist (IL-1Ra) is of clinical interest. Our lab previously reported that chronic exposure to exogenous IL-1 can select for cancer cells that evolve insensitivity to IL-1 signaling, thus rendering IL-1-targeting therapies, like IL-1Ra, irrelevant. While immune cells are the primary source of TME IL-1, cancer cells can also produce and secrete IL-1 to engage in autocrine and/or paracrine signaling. In this context, cancer cell exposure to multiple other sources of exogenous IL-1 beyond autocrine production might also amplify extrinsic IL-1 signaling in these cells, but the consequences of sustained amplified IL-1 signaling on the regulation, function, and therapeutic response of these cancer cells need to be explored. Methods: Using the IL-1-secreting 5637 bladder cancer (BlCa) cell line, we generated chronic IL-1 sublines by spiking the growth medium with additional IL-1α or IL-1β chronically for 6 months. Once established, we assessed subline acute IL-1 sensitivity, paracrine signaling and response to IL-1Ra. Results: Following chronic exposure to elevated exogenous IL-1 levels, the 5637 BlCa cell line retains sensitivity to acute IL-1 and IL-1Ra and maintains the ability to induce IL-1-dependent endothelial cell activation, which is reversed with IL-1Ra. These data suggest that for cancer cells that engage in cell autonomous IL-1 signaling, sustained extrinsic IL-1 exposure does not dampen IL-1 or IL-1Ra sensitivity, supporting the context-dependent use of IL-1 antagonists as rational therapeutics in both acute and chronic inflammatory TMEs.

## 1. Introduction

Bladder cancer (BlCa) is one of the most commonly diagnosed cancers, ranking 10th worldwide, and is the fourth leading cancer in men [1,2]. There is a 71% 5-year survival rate if confined to the bladder; however, this survival rate is reduced to 39% or 8% for regional or distant metastasis, respectively [3]. Hence, it is imperative to understand the underlying mechanisms that lead to the progression of BlCa metastasis.

The tumor microenvironment (TME) is a dynamic niche replete with inflammatory factors secreted by various infiltrating immune cells that serve to eliminate cancer cells—making acute inflammation anti-tumorigenic. However, if acute inflammation persists, then chronic inflammation will ensue and present cancerous cells the opportunity to foster an environment that promotes and maintains inflammation, tumor cell proliferation, cell death evasion, angiogenesis, metastasis, and immunosuppression [4]. Chronic inflammation is sustained and mediated by inflammatory cells that release proinflammatory cytokines, namely interleukin-1 (IL-1) [5,6]. As such, IL-1 is an integral contributor of pro-tumorigenic and malignant behavior that cancer cells acquire from persistent inflammation [7,8].

The IL-1 family includes IL-1 alpha (IL-1α) and IL-1 beta (IL-1β), which are two distinct and major biologically active IL-1 ligands that are essential in mediating inflammation [9]. Additionally, IL-1 receptor antagonist (IL-1Ra) is a well-known regulator of IL-1-induced inflammation that competes with IL-1α and IL-1β to bind IL-1 receptor type I (IL-1RI), rendering the downstream pathway inert. When IL-1α or IL-1β binds to IL-1RI, this interaction elicits the activation of the canonical nuclear factor kappa-light-chain-enhancer of activated B cells (NFκB) signaling pathway [9]. Normally, this signaling cascade results in the heterodimerization of the p50/p65 transcription factor, its nuclear translocation, and the transactivation of target genes. Said target genes are involved in maintaining a well-orchestrated immune and inflammatory response that influences processes involved in cell proliferation, apoptosis, and differentiation [10]. However, the dysregulation of IL-1 and its resulting contribution has been strongly affiliated with the survival and progression of various cancer types, including BlCa [5,7]. This is due to the resulting abnormal NFκB activity which is known to promote tumor cell survival, proliferation, invasiveness, and persistent cytokine and chemokine production, thereby exacerbating the inflammatory milieu [5,7]. Moreover IL-1 drives disease progression by enhancing mechanisms in immune and stromal cells that drive inflammation, angiogenesis, and immunosuppression. This results in tumor-promoting behaviors, including proliferation, migration, and invasion [4,7]. For this reason, IL-1-targeted therapies, like the IL-1 receptor antagonist, anakinra, either alone or in combination with other therapeutic agents, may serve as an ideal treatment in mitigating IL-1-mediated tumorigenesis [8].

Chronic inflammation is affiliated with a fifth of all human cancers [5]. BlCa is one of several cancers that is associated with chronic inflammation and utilizes IL-1 to facilitate pro-tumorigenic interactions with other cells in the TME, for example, by recruiting and activating endothelial cells and fibroblasts to support cancer cell invasion and migration [11,12]. Clinically, IL-1β expression is higher in patients with high-grade and invasive bladder tissue, while low levels of *IL-1RN* mRNA, which encodes for IL-1Ra, correlate with migration and tissue invasion [13,14].

Our lab has previously demonstrated that when prostate cancer cells (PCa) are chronically exposed to IL-1, the cells evolve pro-tumorigenic characteristics that enhance their survival and treatment resistance, including evolving insensitivity to acute IL-1-induced cytotoxicity and attenuated IL-1 intracellular signaling that renders IL-1Ra ineffective [15,16]. Notably, our studies in human prostate and breast cancer cell lines (LNCaP, C4-2, MDA-PCa-2b, MCF7 and T47D) revealed that IL-1 insensitivity is a conserved acquired response to chronic IL-1 exposure [15,16,17,18]. Notably, these cell lines do not produce or secrete IL-1 [19,20]; therefore, in the context of the TME, immune cells would be the primary source of sustained extrinsic IL-1 modulating cancer cell behavior [21]. Cancer cells can, however, produce and secrete IL-1 to engage in IL-1 autocrine and/or paracrine signaling [11], leading us to question whether IL-1-secreting cancer cells would evolve a similar loss of IL-1 and IL-1Ra sensitivity due to extrinsic sources of chronic TME IL-1. In this study, to address this question and better understand how chronic inflammation shapes the TME and therapeutic resistance, we use the grade II non-muscle-invasive human 5637 BlCa cell line as a model to investigate whether cells that constitutively produce and secrete their own IL-1 would evolve the loss of IL-1 and IL-1Ra sensitivity in response to a sustained spike in exogenous IL-1.

## 2. Materials and Methods

### 2.1. Cell Culture

The human bladder cancer cell lines 5637 (ATCC, Manassas, VA, USA; HTB-95637), T24 (ATCC, Manassas, VA, USA; HTB-4), and SW780 (ATCC, Manassas, VA, USA; CRL-2169) and the LNCaP human prostate cancer cell line (ATCC, Manassas, VA, USA; CRL-1740) were maintained in a 37 °C, 5.0% (*v/v*) CO_2_ growth chamber, cultured in Dulbecco Modified Eagle Medium (DMEM (Gibco/Thermo Scientific; Waltham, MA, USA; 1185-092)) supplemented with 10% (*v/v*) fetal bovine essence (FB Essence (FBE); Seradigm, Radnor, PA, USA; 3100-500), 0.4 mM L-glutamine (L-glut; Gibco/Invitrogen, Waltham, MA, USA; 25030-081), and 10 U/mL penicillin G sodium and 10 mg/mL streptomycin sulfate (pen-strep; Gibco/Invitrogen Waltham, MA, USA; 15140-122). The human umbilical vein endothelial cell (HUVEC) line (ATCC, Manassas, VA, USA; PCS-100-010) was maintained in a 37 °C, 5.0% (*v/v*) CO_2_ growth chamber, cultured in Vascular Cell Basal medium (ATCC, Manassas, VA, USA; PCS-100-030) supplemented with Endothelial cell growth kit-BBE (ATCC, Manassas, VA, USA; PCS-100-040) and 10% (*v/v*) fetal bovine essence (FB Essence (FBE); Seradigm, Radnor, PA, USA; 3100-500).

### 2.2. Chronic IL-1 Subline Generation and Maintenance

To generate chronic IL-1 sublines, the 5637 cell line (ATCC, Manassas, VA, USA; HTB-95637) was maintained in DMEM/10% FB Essence (FBE) containing 0.5 ng/mL IL-1α (Gold Bio, St. Louis, MO, USA; 1110–01A-10) or IL-1β (Gold Bio, St. Louis, MO, USA; 1110–01B-10) for 6 months and termed the 5637 IL-1α subline (5637αs) and 5637 IL-1β subline (5637βs), respectively. During subline generation and expansion, 5637 cells cultured in vehicle control (0.1% BSA in 1× PBS) alongside the sublines were termed 5637 parental cells. Following chronic IL-1 exposure, sublines were removed from IL-1-supplemented medium and maintained in normal growth medium. For the phenotypes shown in this report, sublines were moved to and cultured in normal growth medium for at least 3 months to ensure stable subline phenotypes. IL-1 concentration, treatment times, and maintenance are based on previously reported sublines generated in other cell line backgrounds [15,16,18]. Cell line authentication was performed for 5637 parentals, 5637αs, and 5637βs with STR profiling by the DNA Genotyping Core, University of Texas Southwestern Medical Center, TX, USA. We hereby confirm that none of the cell lines used require any ethical approval for their use.

### 2.3. Cell Treatments

#### 2.3.1. Cytokines

Human recombinant IL-1α (GoldBio, St. Louis, MO, USA; 1110-01A-100), IL-1β (GoldBio, St. Louis, MO, USA; 1110-01B-100), and IL-1Ra (Goldbio, St. Louis, MO, USA; 1110-01C) were resuspended in 0.1% bovine serum albumin (BSA) (Thermo Fisher Scientific, Waltham, MA, USA; BP 1600-1) in 1X phosphate-buffered saline (PBS; Corning, Manassas, VA, USA; 21-040-CM). Cells were treated with vehicle control (0.1% BSA in 1X PBS), IL-1 or IL-1Ra added to DMEM/10% FBE growth medium.

#### 2.3.2. Conditioned Medium (CM)

In this study, 5637, 5637αs, and 5637βs cells were grown in DMEM/10% FBE for 4 days, and the conditioned medium was subsequently collected and filtered (EMD Millipore, Burlington, MA, USA; SCGPU05RE; pore size 0.22 μm) to remove cell debris.

### 2.4. Protein Analysis

#### 2.4.1. Western Blot

Protein isolation and Western blot were conducted as previously published [15]. Primary antibodies: LCN2 (Cell Signaling Technology, Danvers, MA, USA; 44058S), CXCL1/2 (Cell Signaling Technology, Danvers, MA, USA; 24376T), IL-6 (Cell Signaling Technology, Danvers, MA, USA; 12153S), IL-8 (Cell Signaling Technology, Danvers, MA, USA; 94407S), AR (Cell Signaling Technology, Danvers, MA, USA; D6F11), PARP (Cell Signaling Technology, Danvers, MA, USA; 9532S), SOD2 (Cell Signaling Technology, Danvers, MA, USA; 1314S),), β-actin (Santa Cruz, Santa Cruz, CA, USA; sc-69879), PSA (Cell Signaling Technology, Danvers, MA, USA; 5365S), NKX3.1 (Cell Signaling Technology, Danvers, MA, USA; D2Y1A). Secondary antibodies: Sheep anti-mouse (Jackson ImmunoResearch Laboratories, Grove PA, USA; 515-035-062) and goat anti-rabbit (Abnova, Walnut, CA, USA; PAB10822).

#### 2.4.2. Immunostaining and Microscopy

Cells were plated into a 48-well plate (Nunclon, Roskilde, Denmark; 150687). Cells were subsequently fixed with 4% paraformaldehyde at room temperature for 20 min or fixed and permeabilized with 100% methanol at −20 °C for 30 min. Fixed cells were blocked with 2.5% BSA in 1× PBS at room temperature for at least 30 min. Antibodies were diluted in 2.5% BSA in 1× PBS. Cell nuclei were stained with DAPI (Roche Diagnostics, North Ryde, NSW, Australia; 10236276001). Primary antibodies: β-catenin (Cell Signaling Technology, Danvers, MA, USA; 9562S), AR (Cell Signaling Technology, Danvers, MA, USA; D6F11), and Ki-67 (Millipore, Darmstadt, Germany, MAB4190. Secondary antibodies: goat anti-rabbit (Invitrogen, Waltham, MA, USA) and goat anti-mouse (Invitrogen, Waltham, MA, USA; A32723). Immunostained cells were imaged at ×10 magnification using the Nikon Eclipse Ti fluorescence microscope and Nikon NIS Elements software Version 5 41 02 (Nikon, Melville, NY, USA).

#### 2.4.3. Enzyme-Linked Immunosorbent Assay (ELISA)

A total of 10,000 cells were seeded into a 6-well plate and cultured in serum-free DMEM for 48 h. Conditioned medium was subsequently collected and filtered (EMD Millipore, Burlington, MA, USA; SCGPU05RE; pore size 0.22 μm) to remove cell debris. Conditioned medium was assayed for relative cytokine levels using the Human Cytokine ELISA Plate Array I (Colorimetric) according to the manufacturer’s instructions (Signosis Inc., Santa Clara, CA, USA; EA4002). Colorimetric optical density (450 nm) was read using the Cytation3 Cell Imaging Multi-Mode Reader (BioTek, Winooski, VT, USA).

### 2.5. RNA Isolation and Reverse Transcription Quantitative Polymerase Chain Reaction (RT-qPCR)

Total RNA was extracted from the cells using the GeneJET RNA Purification Kit according to the manufacturer’s instructions (Thermo Scientific, Waltham, MA, USA; K0732). cDNA was generated using iScript Reverse Transcription Supermix (Biorad, Hercules, CA, USA; 170-8841). The RT-QPCR was prepared using iTaq Universal SYBR Green Supermix (Biorad, Hercules, CA, USA; 172-5125). Primer sequences for the genes of interest are listed below. Gene of interest cycle times (CTs) were normalized to β-actin. Relative mRNA levels were calculated using the 2−ΔΔCT method. Primer sequences—5′-3′: *Mitochondrial Superoxide Dismutase 2 (SOD2*), forward GGCCTACGTGAACAACCTGA, reverse GTTCTCCACCACCGTTAGGG; *chemokine ligand 1 (CXCL1)*, forward CCCCAAGAACATCCAAAGTGTG, reverse ATGCAGGATTGAGGCAAGC; *chemokine ligand 2 (CXCL2)*, forward AGCTTGTCTCAACCCCGC, reverse CAGTTGGATTTGCCATTTTTCAGCA; *interleukin-6 (IL-6)*, forward AGTGAGGAACAAGCCAGAGC, reverse ATTTGTGGTTGGGTCAGGGG; *interleukin-8 (IL-8)*, forward ACACTGCGCCAACACAGAAAT, reverse AACTTCTCCACAACCCTCTGC; *intracellular adhesion molecule 1 (ICAM)*, forward AGCTTCGTGTCCTGTATGGC, reverse TTTTCTGGCCACGTCCAGTT; *beta actin (β-actin)*, forward GATGAGATTGGCATGGCT TT, reverse CACCTTCACCGGTCCAGTTT; *interleukin-1α (IL-1α)*, forward GTAGCAACCAACGGGAAGGT, reverse AAGGTGCTGACCTAGGCTTG; *interleukin 1β*, forward CAGAAGTACCTGAGCTCGCC, reverse AGGTCCTGGAAGGAGCACTT; *interleukin-1 receptor type 1 (IL-1R1)*, forward TGGGGAAGGGTCTACCTCTG, reverse TCCCCAACGTAGTCATCCCT.

### 2.6. Cell Cluster Nucleus Distance

The distance between DAPI-stained cells within a visually defined cluster was determined by using Image J 1.54t (National Institutes of Health, Bethesda, MD, USA). One cell in the cluster was used as the reference cell, and the distance from the reference cell for each of the other cells in the cluster was measured in pixels. *n* ≥ 13 clusters of cells were analyzed per treatment. The standard deviation within a cluster (red bar in graph) and the pooled standard deviation (table) within a treatment were calculated using Microsoft Excel. Pooled standard deviation was calculated as described in [22]. The smaller the standard deviation, the more compact the cells are in the cluster. The larger the standard deviation, the more dispersed the cells are in the cluster.

### 2.7. Statistical Analysis

Statistical significance was determined utilizing an unpaired Student’s *t*-test conducted in Microsoft Excel. *p*-values ≤ 0.05 were considered to be statistically significant and denoted by asterisks (* *p* ≤ 0.05; ** *p* ≤ 0.05; *** *p* ≤ 0.005). “NS” indicates no statistical significance. Error bars indicate ± standard deviation (SD) where *n* ≥ 3 biological replicates. Experiments were repeated three times, and representative data are shown.

## 3. Results

### 3.1. Sensitivity Is Shown by 5637 BlCa Cells After a Spike in Exogenous IL-1

**Figure 1:**
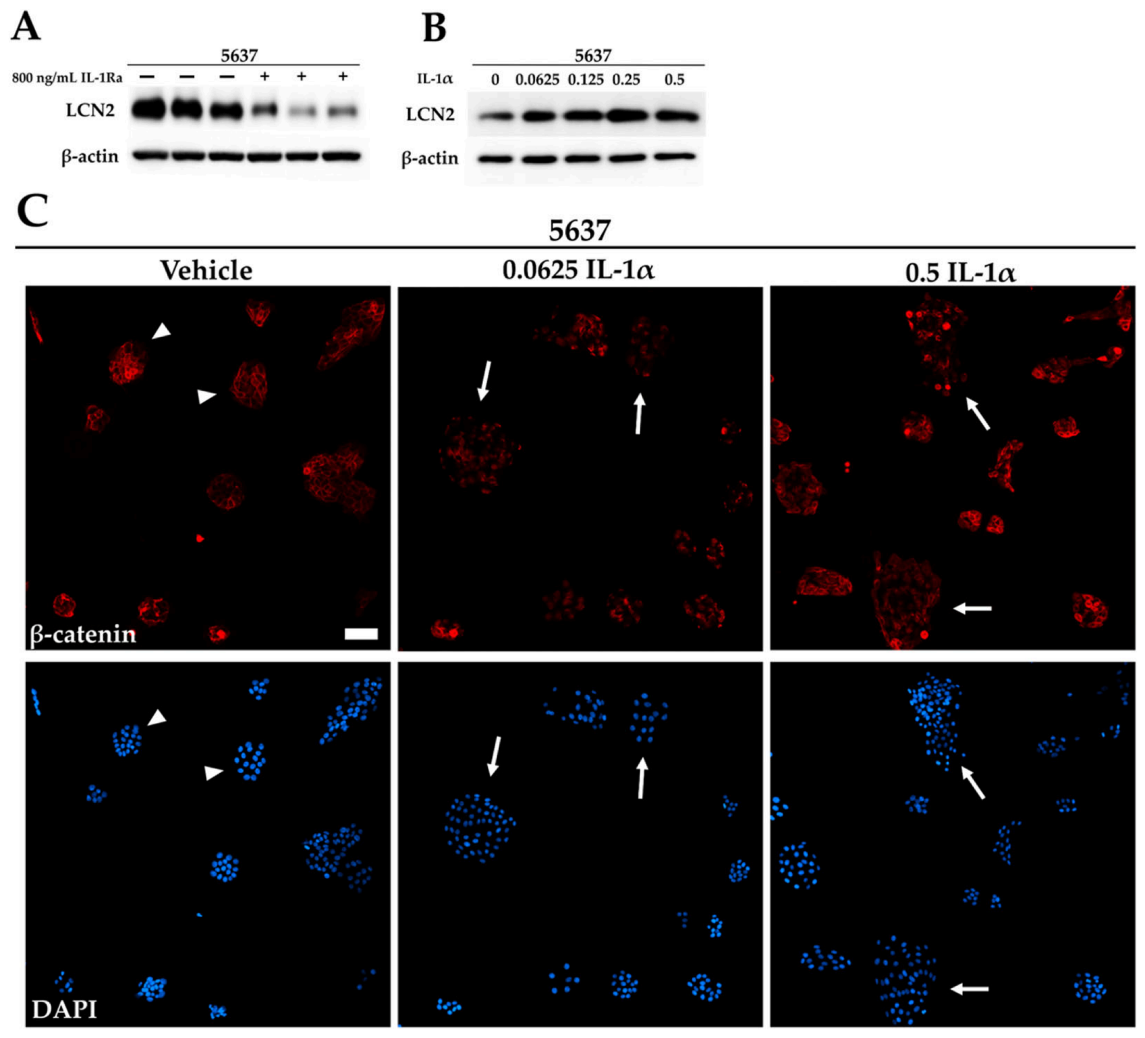
5637 BlCa cells respond to a spike in exogenous IL-1 levels. In this study, 5637 cells were treated with (**A**) 800 ng/mL IL-1Ra for 4 days (3 biological replicates shown) or (**B**) 0.0625 ng/mL, 0.125 ng/mL, 0.25 ng/mL, or 0.5 ng/mL IL-1α and analyzed by Western blot for the canonical IL-1-induced gene, *lipocalin 2 (LCN2)*. (**C**) Moreover, 5637 cells were treated with 0.0625 ng/mL, 0.125 ng/mL, 0.25 ng/mL, or 0.5 ng/mL IL-1α for 3 days; fixed; immunostained for the cell–cell adhesion protein, β-catenin (Texas Red); and stained with the nuclear stain, DAPI. Cells were imaged at 10× magnification, scale bar = 100 μm. LCN2 levels were reduced in cells treated with IL-1Ra alone, indicating IL-1 autocrine signaling. In response to the addition of exogenous IL-1α to the medium, LCN2 levels were induced, and the rounded cell clusters found in control growth medium became more dispersed and irregular-shaped, indicating IL-1 sensitivity beyond autocrine signaling. β-actin is the Western blot loading control. Solid arrowheads indicate representative compact, round clusters. Arrows indicate representative irregular organization and dispersion of cells in a cluster.

Previous studies have shown IL-1β to be among the various factors the grade II human BlCa cell line, 5637, constitutively secretes [23]. Thus, we first investigated whether 5637 BlCa cells participate in IL-1 autocrine signaling. We treated 5637 cells with 800 ng/mL of IL-1Ra for 4 days and monitored the protein levels of the IL-1 target gene, *lipocalin-2 (LCN2)*, via Western blot as a surrogate for IL-1 activity. Exogenous IL-1Ra reduces basal LCN2 accumulation, indicating IL-1 autocrine activity in the 5637 BlCa cell line (Figure 1A).

Since IL-1 is simultaneously produced by multiple cell types in the TME, we determined whether 5637 cells could respond to additional exogenous IL-1 beyond autocrine signals. IL-1α and IL-1β both bind the IL-1 receptor and elicit similar biological responses [24], so dose experiments were conducted with IL-1α only. Thus, 5637 cells were treated with increasing doses of IL-1α (0.0625 ng/mL, 0.125 ng/mL, 0.25 ng/mL, 0.5 ng/mL) acutely for 3 days and analyzed for LCN2 accumulation by Western blot. LCN2 accumulation is induced at each IL-1 dose, indicating that 5637 cells do respond to the additional IL-1α (Figure 1B). The immunofluorescence imaging of β-catenin-stained cells, counterstained with DAPI nuclear stain, reveals that the additional IL-1α induces cell dispersion and irregularly shaped cell clusters showing some loss of cell–cell adhesion (Figure 1C). Taken together, we see that while 5637 cells secrete IL-1 and participate in autocrine signaling, they do respond to additional IL-1 levels, further suggesting that cancer cell IL-1 autocrine signaling is amplified by TME IL-1 paracrine signals.

Notably, compared to the 5637 cells, the grade I human BlCa cell line, SW780, and the IL-1-secreting grade III human BlCa cell line, T24 [25], do not show an appreciable response to 50 ng/mL acute IL-1 treatment (Figure S1). Thus, the 5637 BlCa cell line is a unique model for investigating the molecular and cellular consequences of exogenous chronic IL-1 exposure in cells that also secrete IL-1.

### 3.2. Sensitivity to Acute IL-1 and IL-1Ra Is Retained by 5637 Chronic IL-1 Sublines

**Figure 2:**
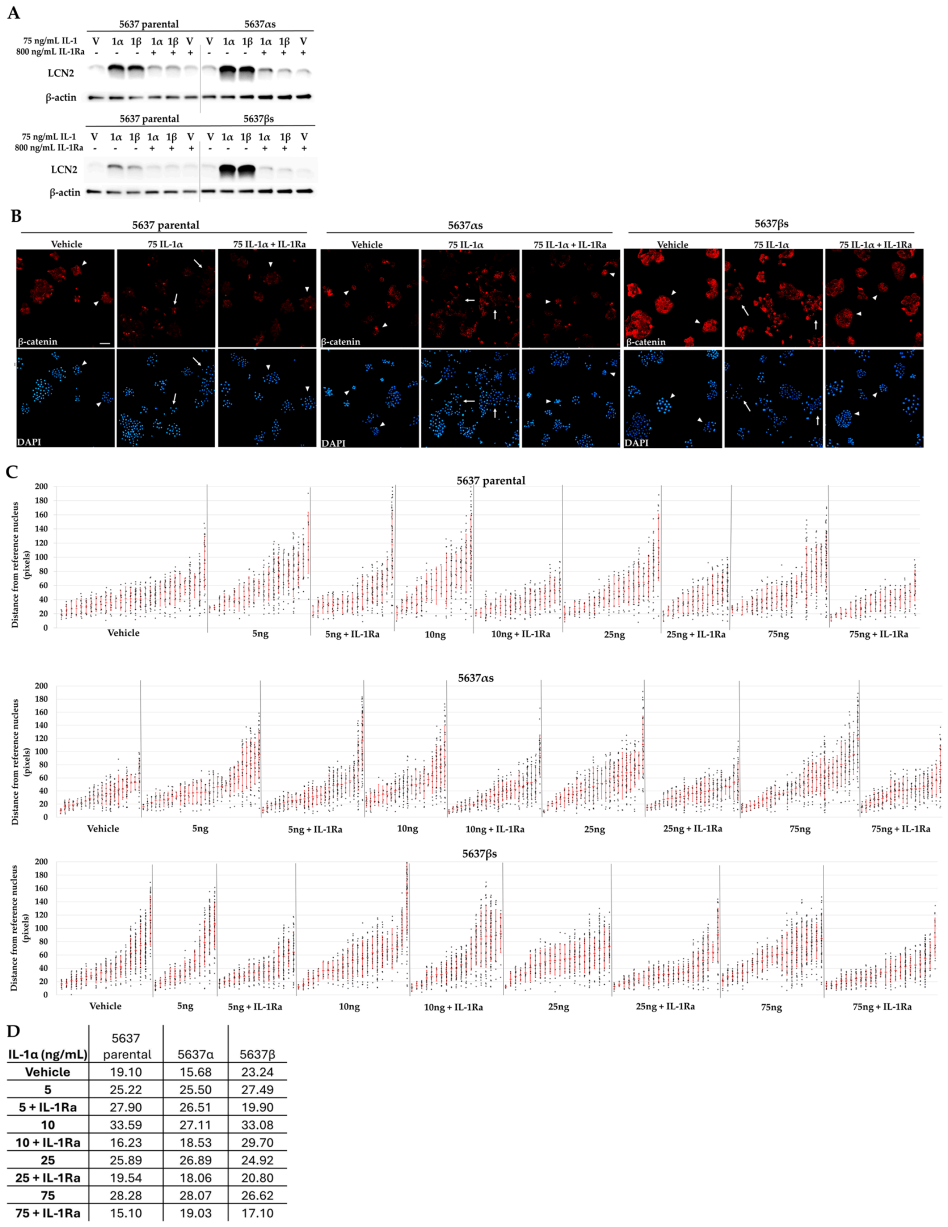
5637 chronic IL-1 sublines retain sensitivity to acute IL-1 and IL-1Ra. Parental cells (5637 parental) and chronic IL-1 sublines (5637αs, 5637βs) were (**A**) treated acutely for 3 days with 75 ng/mL IL-1α or IL-1β ± 800 ng/mL IL-1Ra and analyzed for LCN2 accumulation by Western blot or (**B**–**D**) treated acutely for 3 days with 0.5 ng/mL, 5 ng/mL, 10 ng/mL, 25 ng/mL, or 75 ng/mL of IL-1α ± 800 ng/mL IL-1Ra; fixed; immunostained for the cell–cell adhesion protein, β-catenin (Texas Red); and stained with the nuclear stain, DAPI. In both parental and chronic IL-1 subline cells, IL-1 induced LCN2 accumulation (**A**) and irregular-shaped clusters of dispersed cells (**B**–**D**) that are attenuated by IL-1Ra. β-actin is the Western blot loading control. Cells were imaged at 10× magnification, scale bar = 100 μm. Solid arrowheads indicate representative compact, round clusters. Arrows indicate representative irregular, dispersed organization of cells in a cluster. The standard deviation within a cluster is indicated by the red bar in the graph (**C**), and the pooled standard deviation of a treatment is indicated in the table (**D**).

We previously reported that chronic IL-1 selects for cells that evolve IL-1 insensitivity [15,16,17,18]. Given that 5637 cells do respond to acute spikes in exogenous IL-1 levels (Figure 1), we wanted to determine whether chronic exposure would render 5637 cells IL-1-insensitive. To do so, 5637 cells were cultured in 0.5 ng/mL IL-1α or IL-1β for 6 months, followed by growth for at least 3 months in normal growth medium to establish stable chronic sublines labeled, respectively, 5637αs and 5637βs. During subline generation, 5637 cells were cultured in vehicle control alongside the sublines to establish the parental cell line control, labeled 5637 parental. To determine whether the 5637 chronic sublines acquired IL-1 insensitivity, 5637 parental cells and the chronic IL-1 sublines were treated with 75 ng/mL IL-1α or IL-1β acutely for 3 days in the absence or presence of IL-1Ra. LCN2 Western blot shows that IL-1 induces LCN2 accumulation, which is blocked by IL-1Ra, in parental and subline cells (Figure 2A). Cells were also treated with increasing concentrations of IL-1α (0.5 ng/mL, 5, ng/mL, 10 ng/mL, 25 ng/mL, and 75 ng/mL) in the absence or presence of IL-1Ra. Fluorescence imaging (Figure 2B) and the quantification of cluster cell dispersion (Figure 2C,D) show that IL-1 induces irregularly shaped clusters with dispersed cells showing some loss of cell–cell adhesion in both parental and chronic IL-1 subline cells. This morphology is attenuated by IL-1Ra. Thus, 5637 cells chronically exposed to IL-1 retain sensitivity to IL-1 and IL-1Ra.

As stated above, we previously reported that chronic IL-1 selects for cells that evolve IL-1 insensitivity [15,16,17,18]. In particular, the LNCaP prostate cancer (PCa) cell line shows significant attenuation of IL-1 signaling following chronic IL-1 exposure [15]. We compared the acute IL-1 response of 5637 parental and chronic IL-1 subline cells (5637αs, 5637βs) to the LNCaP parental and chronic IL-1 subline cells (LNas1, LNbs1) treated, respectively, with 50 and 25 ng/mL IL-1 for three days and assayed for *LCN2* mRNA levels as a surrogate for IL-1 intracellular signaling (Figure S2A). LNCaP parental cells show significantly higher acute IL-1 response than 5637 parental cells, exhibiting a >150-fold increase in *LCN2* levels versus a 1.3-fold increase, respectively (Figure S2A). Furthermore, while chronic IL-1 exposure selects for LNCaP cells (i.e., LNas1, LNbs1) that evolve starkly attenuated IL-1 sensitivity, the chronic IL-1 5637 subline cells (i.e., 5637αs, 5637βs) retain an IL-1 response comparable to 5637 parental cells (Figure S2A). One possible mechanism that may underlie 5637 and LNCaP differential response to acute and chronic IL-1 is IL-1 cytokine and receptor levels. Compared to LNCaP cells, 5637 cells have higher basal *IL-1α* and *IL-1β* mRNA levels and lower basal *IL-1R1* receptor levels (Figure S2B). Thus, 5637 cells are primed to secrete and facilitate IL-1 paracrine signaling, while LNCaP cells are primed to respond to exogenous IL-1. Compared to LNCaP cells, T24 and SW780 BlCa cell lines show similar *IL-1* and *IL-1R1* expression patterns as 5637 cells, but unlike 5637 cells, T24 and SW780 cells do not respond to our exogenous IL-1 treatment conditions (Figure S1), suggesting that other or additional mechanisms downstream of the IL-1 receptor/ligand interaction (e.g., transcriptional regulators) control relative IL-1 sensitivity and response.

### 3.3. Parental and Chronic IL-1 Sublines Show Comparable Cytokine Secretome and IL-1-Dependent Paracrine Signaling

**Figure 3:**
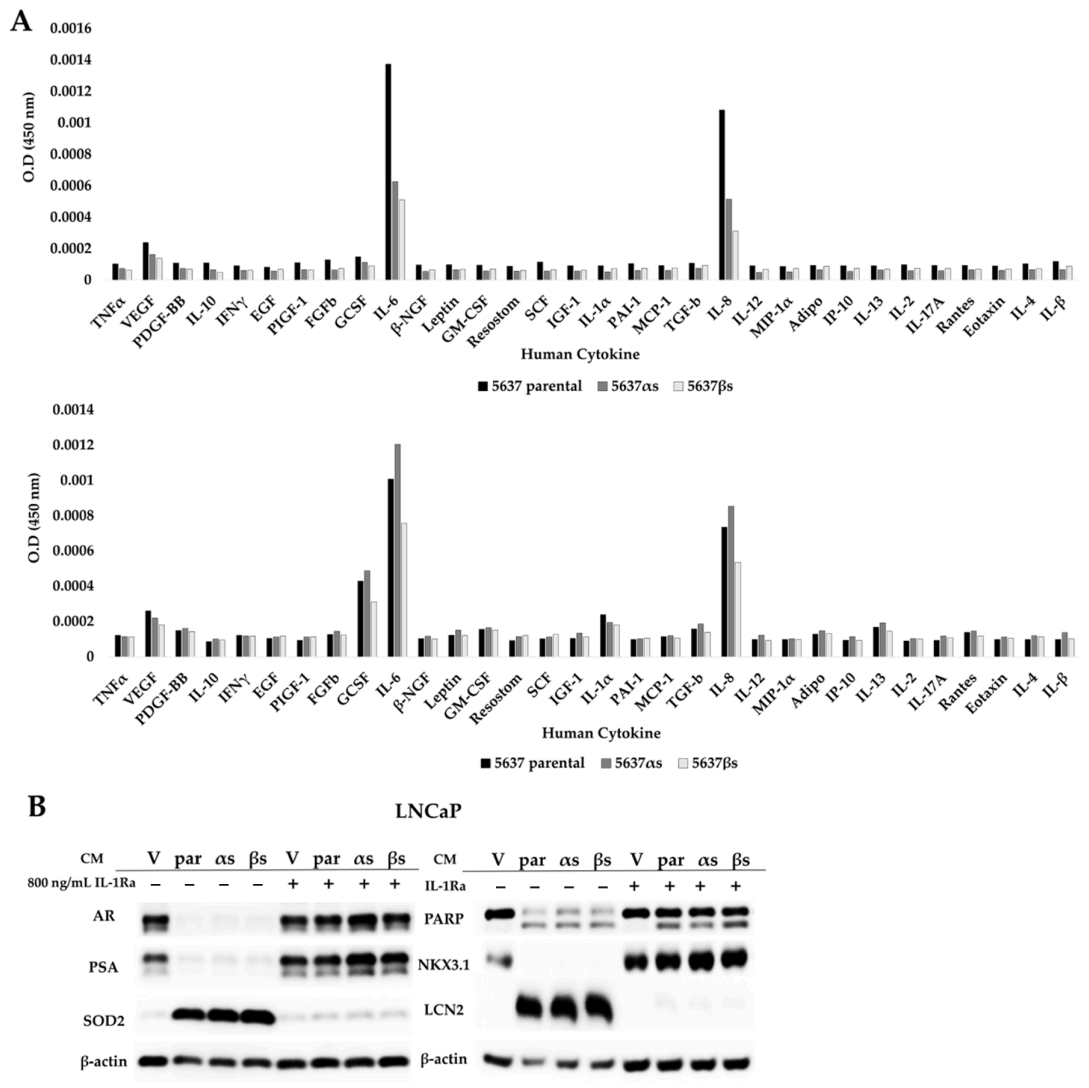
Parental and chronic IL-1 sublines show comparable cytokine secretome and IL-1-dependent paracrine signaling. (**A**) Cells were plated in low serum (2.5% FBS) for 46 h and subsequently grown in serum-free media for 48 h. Conditioned medium (CM) was collected and incubated on a cytokine array plate for 2 h, and colorimetric optical density (O.D.) was measured at 450 nm. The ELISA experiment was conducted twice. (**B**) The LNCaP PCa cell line was treated acutely with non-conditioned growth medium (DMEM/10% FBE, vehicle), 5637 parental CM, 5637αs CM, or 5637βs CM ± 800 ng/mL IL-1Ra for 2 days and analyzed by Western blot for AR and AR target genes, *PSA* and *NKX3.1*; canonical IL-1 target genes, *SOD2* and *LCN2*; and PARP cleavage. Parental and subline cells show comparable secretomes and IL-1-dependent paracrine regulation of canonical AR and IL-1 signaling and apoptosis in LNCaP cells. β-actin is the Western blot loading control.

Notably, 5637 BlCa cells have been previously reported to be strong paracrine signalers, secreting a number of bioactive cytokines, including IL-1β [23]. Therefore, we sought to interrogate whether chronic IL-1 exposure alters 5637 secretome and IL-1 paracrine signaling. We collected conditioned media (CM) derived from 5637 parental, 5637αs, or 5637βs cells and qualitatively analyzed the secretory profile of various cytokines, including IL-1α and IL-1β. The ELISA experiment was performed twice and shows no reproducible, appreciable difference in parental and subline cytokine secretomes (Figure 3A).

As stated earlier, with relatively low *IL-1α* and *IL-1β* expression and relatively high *IL-1R1* expression, the LNCaP PCa cell line is highly sensitive to exogenous IL-1 (Figure S2A,B), making LNCaP cells a valuable proof-of-concept model for interrogating 5637-mediated IL-1 paracrine signaling. To assess IL-1-dependent 5637 paracrine signaling, we treated IL-1-sensitive LNCaP PCa cells in the absence or presence of IL-1Ra with conditioned medium (CM) derived from 5637 parental, 5637αs, or 5637βs cells for 2 days. Western blot shows that CM from 5637 parental, 5637αs, or 5637βs cells induces the repression of androgen receptor (AR) and AR target genes, *PSA* and *NKX3.1*; induction of canonical IL-1 target genes, *SOD2* and *LCN2*; and induction of apoptosis (PARP cleavage), which are attenuated by IL-1Ra in LNCaP cells (Figure 3B). In addition, immunostaining shows that CM from 5637 parental, 5637αs, or 5637βs cells represses AR and Ki67 nuclear accumulation, indicating the downregulation of AR activity and proliferation, respectively (Figure S2C). CM-mediated repression is reversed by IL-1Ra (Figure S2C). Thus, CM from 5637 parental, 5637αs, or 5637βs cells activate IL-1-dependent molecular and cellular responses in LNCaP cells. Taken together, 5637 cells chronically exposed to IL-1 maintain cytokine secretion and IL-1-dependent paracrine signaling.

### 3.4. Chronic IL-1 Sublines Retain the Ability to Activate HUVECs in an IL-1-Dependent Manner

**Figure 4:**
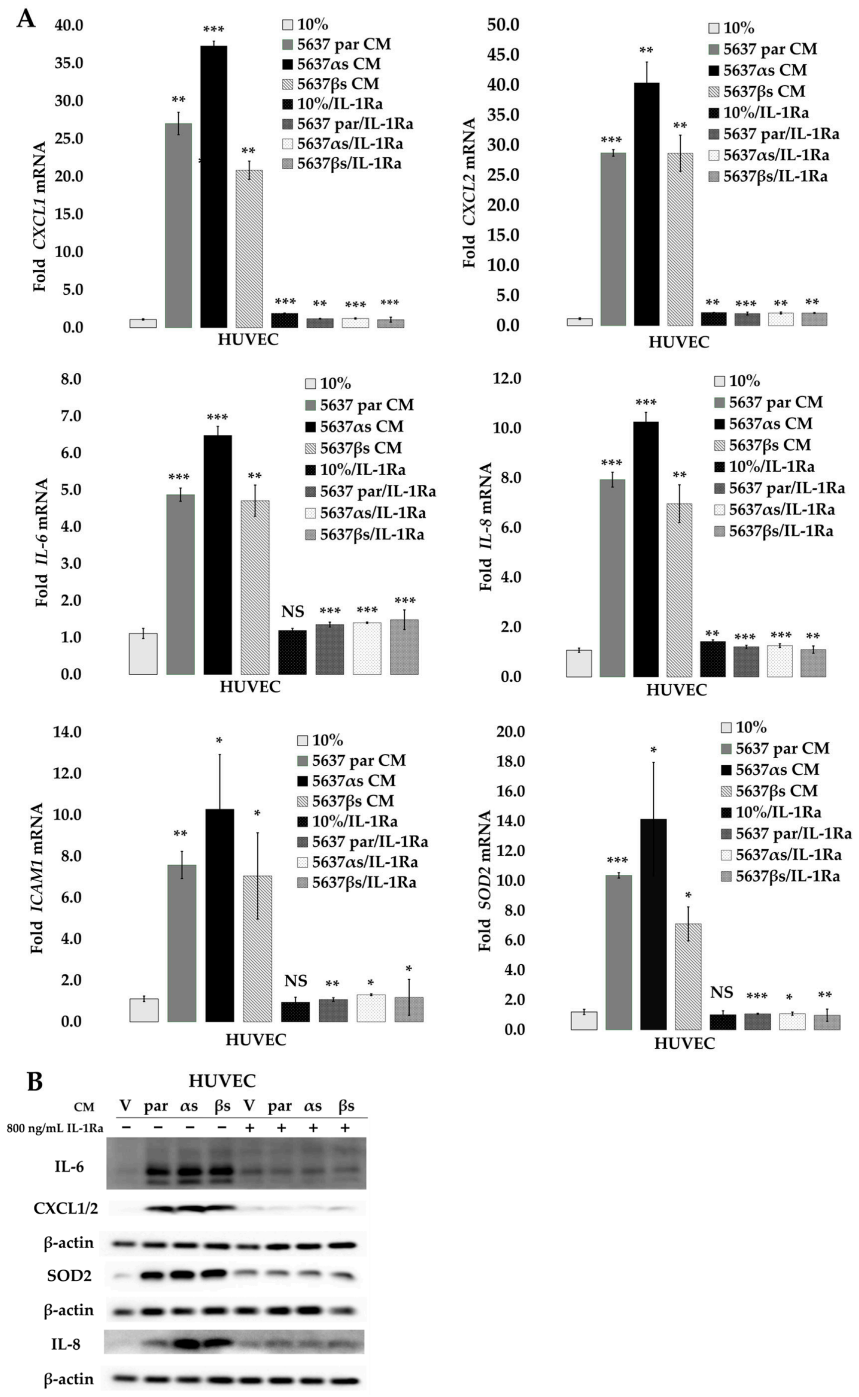
Chronic IL-1 sublines retain the ability to activate HUVEC endothelial cells. HUVEC cellss were treated acutely with control media (V), 5637 parental CM, 5637αs CM, or 5637βs CM ± 800 IL-1Ra for 2 days and analyzed for (**A**) RNA (RT-qPCR) or (**B**) protein accumulation (Western blot) for canonical IL-1 target genes, *LCN2* or *SOD*, and endothelial cell activation marker genes, *chemokine ligand 1 (CXCL1)*, *chemokine ligand 2 (CXCL2)*, *interleukin-6 (IL-6)*, *interleukin-8 (IL-8)*, and *Intercellular Adhesion Molecule 1 (ICAM1)*. CM from both parental and chronic IL-1 sublines induces ECA markers and IL-1 target genes in HUVECs that are attenuated by IL-1Ra. RT-qPCR, *n* = 3 biological replicates; error bars = +/−STDEV; *p*-value = * ≤ 0.05, ** ≤ 0.005, *** ≤ 0.0005. *p*-values are shown for the CM-only treatment compared to DMEM/10% FBE control medium and for the IL-1Ra treatment compared to CM alone. Fold mRNA levels are normalized to the vehicle control.

Endothelial cells are one of the various cell types found to shape the TME [4]. Paracrine crosstalk between endothelial cells and tumor cells prompt endothelial cell activation (ECA), a process in which the vascular endothelium is converted to a cell-adhesive state which, in turn, facilitates the binding of immune cells [26]. IL-1 is a known factor in this paracrine interplay and aids in creating a proinflammatory surface that drives metastasis by promoting immune cell recruitment, compromising the endothelial barrier, and enabling vascular adhesion and transendothelial migration of tumor cells, including in the context of BlCa cell paracrine signaling [25,26]. Thus, we set out to investigate how chronic IL-1 influences the paracrine interaction between 5637 BlCa and endothelial cells. We exposed human umbilical vein endothelial cells (HUVECs) to 5637 parental and chronic IL-1 subline CM in the absence or presence of IL-1Ra for 2 days to determine parental and sublines’ ability to induce IL-1-mediated ECA. In addition to monitoring the IL-1 induction of SOD2, we used RT-qPCR (Figure 4A) and Western blot (Figure 4B) analyses to assess known ECA markers—specifically *chemokine ligand 1 (CXCL1)*, *chemokine ligand 2 (CXCL2)*, *interleukin-6 (IL-6)*, *interleukin-8 (IL-8)*, and *Intercellular Adhesion Molecule 1 (ICAM1)*. In response to the CM from 5637 parental and chronic IL-1 sublines, we observed a significant increase in SOD2 and the ECA markers. This induction is attenuated by IL-1Ra, demonstrating that HUVEC activation is IL-1-mediated (Figure 4). Thus, chronic IL-1 exposure does not alter 5637 IL-1-mediated ECA.

## 4. Discussion

Chronic inflammation is essential in modulating the TME, making it a key contributor to tumor initiation and evolution [27]. Because acute inflammation can be cytotoxic and cytostatic for cancer cells, there is selective pressure for cancer cells to evolve resistance to the anti-tumorigenic effects of inflammation when acute inflammation becomes chronic. In accordance with this, our lab has previously shown that while acute IL-1 exposure results in cytotoxic and cytostatic anti-tumorigenic responses in PCa cell lines, chronic IL-1 exposure drives PCa cells to evolve acute IL-1 insensitivity concomitant with acquired pro-tumorigenic phenotypes that IL-1Ra would be ineffective at blocking [15,16,18]. We also observed chronic IL-1-induced IL-1 insensitivity in breast cancer (BCa) cell lines [17], suggesting that the response to chronic IL-1 exposure is conserved and could lead to IL-1Ra therapeutic resistance across cancer types. Therefore, we sought to expand our analysis to other cancer types where IL-1 signaling is functionally and clinically relevant, such as BlCa.

IL-1Ra has been shown by others to effectively block IL-1 autocrine- and paracrine-mediated BlCa invasion and endothelial cell activation (ECA) using the T24 BlCa cell line [14,25]. Differently from these other studies, we use canonical IL-1 target gene expression as a surrogate for IL-1 and IL-1Ra sensitivity, and despite similar acute IL-1 and IL-1Ra treatment conditions, we did not detect changes in T24 IL-1 intracellular response to exogenous IL-1 (Figure S1). Perhaps analyzing canonical IL-1 target genes different from those used in our study would have shown T24 IL-1 sensitivity in our treatment conditions. Notwithstanding, the 5637 BlCa cell line, which produces and secretes IL-1 (Figure 3 and Figure S2), does show sensitivity to spikes in exogenous IL-1 (Figure 1 and Figure 2 and Figure S2), including the upregulation of canonical IL-1 target genes (Figure 1B), the disruption of cell–cell clustering and adhesion (Figure 1C), IL-1-dependent ECA (Figure 4), and IL-1Ra sensitivity (Figure 1 and Figure 2). Thus, the 5637 cell line is a rational model for determining whether chronic spikes in exogenous IL-1 can drive IL-1-producing cells to lose sensitivity to IL-1 and IL-1Ra.

We find that the 5637 BlCa cell line maintains acute IL-1 sensitivity following exogenous chronic IL-1 exposure (Figure 2) and thus maintains sensitivity to IL-1Ra (Figure 2). These results are in contrast to our previous findings for several different breast and prostate cancer cell lines which evolve acute IL-1 insensitivity in response to chronic IL-1 exposure [15,16,17,18]. Unlike the breast and prostate cancer cell lines (Figure S2) [19,20], the 5637 BlCa cell line produces and secretes IL-1 (Figure 3 and Figure S2) and engages in constitutive IL-1 autocrine signaling (Figure 1), resulting in cell-autonomous chronic IL-1 exposure. Thus, there is no apparent selective pressure to attenuate IL-1 signaling even in the presence of additional exogenous IL-1 sources. In contrast, IL-1-sensitive cell lines like LNCaP evolve IL-1 insensitivity because of the selective pressure to evade acute IL-1-induced cytotoxicity and cytostasis, emerging as more fit and treatment-resistant [15,16,18].

A direct comparison of the 5637 and LNCaP cell lines shows that LNCaP cells have a much more robust acute IL-1 response and a more significant chronic IL-1-induced attenuated response than 5637 cells (Figure S2A). The differences in the basal expression of *IL-1α*, *IL-1β*, and *IL-1R1* in LNCaP versus 5637 cell lines is one possible mechanism underlying the cell line-specific response to IL-1, requiring further investigation. LNCaP cells have higher *IL-1R1* expression levels, while 5637 cells show greater *IL-1α* and *IL-1β* expression (Figure S2) and secrete IL-1α and IL-1β (Figure 3). Taken together, receptors are more readily available to bind exogenous IL-1 in LNCaP cells to elicit an acute robust response, while 5637 cells engage in constitutive autocrine signaling that shows comparatively minimal responsiveness to additional IL-1. Additionally, mechanisms downstream of the IL-1 receptor/ligand interaction (e.g., transcriptional regulators) could control relative sensitivity and response to acute and chronic IL-1. Indeed, we find that LNCaP response to chronic IL-1 exposure is dysregulated downstream of the receptor/ligand interaction [15] and shows altered epigenetic regulation (manuscript in preparation).

The TME is replete with IL-1 from multiple sources, including cancer and immune cells, thus potentially amplifying cell-autonomous IL-1 signaling beyond the autocrine response. Indeed, we show that extrinsic IL-1 can amplify IL-1 signaling in 5637 cells beyond the autocrine response to induce phenotypes such as cell–cell disassociation (Figure 1 and Figure 2) but is not sufficient to drive the IL-1 and IL-1Ra insensitivity observed in cancer cells that do not engage in IL-1 autocrine signaling [15,16,17,18]. Thus, dependent on context, IL-1Ra would be an effective therapeutic in the acute and chronically inflamed TME to block IL-1-induced pro-tumorigenic processes such as cell migration and tumor angiogenesis.

Finally, while we did not identify significant molecular or cellular changes induced by chronic IL-1 in 5637 cells, the 5637 chronic IL-1 sublines may have undergone other inherent changes not assessed in this report, including gene expression changes, chromatin remodeling, or changes in intra- and extracellular signaling crosstalk with other growth factors and cytokines. Thus, further studies are required to make conclusions regarding any alterations to the 5637 chronic IL-1 sublines.

## 5. Conclusions

In conclusion, our results demonstrate that when chronically exposed, BlCa cell lines participating in IL-1 autocrine and paracrine signaling retain sensitivity to IL-1, specifically when there is a surge in exogenous levels. Consequently, they retain their sensitivity to IL-1Ra. We find that IL-1Ra prevents the IL-1-induced loss of BlCa cell–cell clustering and adhesion and BlCa IL-1-dependent paracrine activation of endothelial cells—all of which may support tumorigenic phenotypes such as angiogenesis and metastasis. Taken together, our chronic IL-1 BlCa cell line model is a valuable tool for characterizing tumor inflammation and responsiveness to IL-1-targeted therapies in the context of acute and chronic surges in TME IL-1.

## Data Availability

The original contributions presented in this study are included in the article/Supplementary Materials. Further inquiries can be directed to the corresponding author.

## Supplementary Materials

Please provide: The following supporting information can be downloaded at: https://www.mdpi.com/article/10.3390/cells15151376/s1, Figure S1: Human BlCa cell lines, T24 and Sw780, are insensitive to exogenous IL-1; Figure S2: LNCaP cells are more sensitive to acute and chronic IL-1 than 5637 cells.

## Abbreviations

The following abbreviations are used in this manuscript:

BlCa	Bladder cancer
PCa	Prostate cancer
HUVEC	Primary umbilical vein endothelial cell
IL-1α	Interleukin-1 alpha
IL-1β	Interleukin-1 beta
IL-1Ra	Interleukin 1 receptor antagonist
5637α	5637 chronic IL-1α subline
5637β	5637 chronic IL-1β subline
