# Supplementary material for: Bladder Cancer Cells Maintain Paracrine IL-1 Signaling and IL-1Ra Sensitivity Following Chronic IL-1 Exposure
Source: Cells. 2026 Jul 30;15(15):1376. doi: 10.3390/cells15151376 (PMC13465337; doi:10.3390/cells15151376)
Supplement: Supplementary file 1 [file cells-15-01376-s001.zip › cells-4375425-supplementary.pdf]

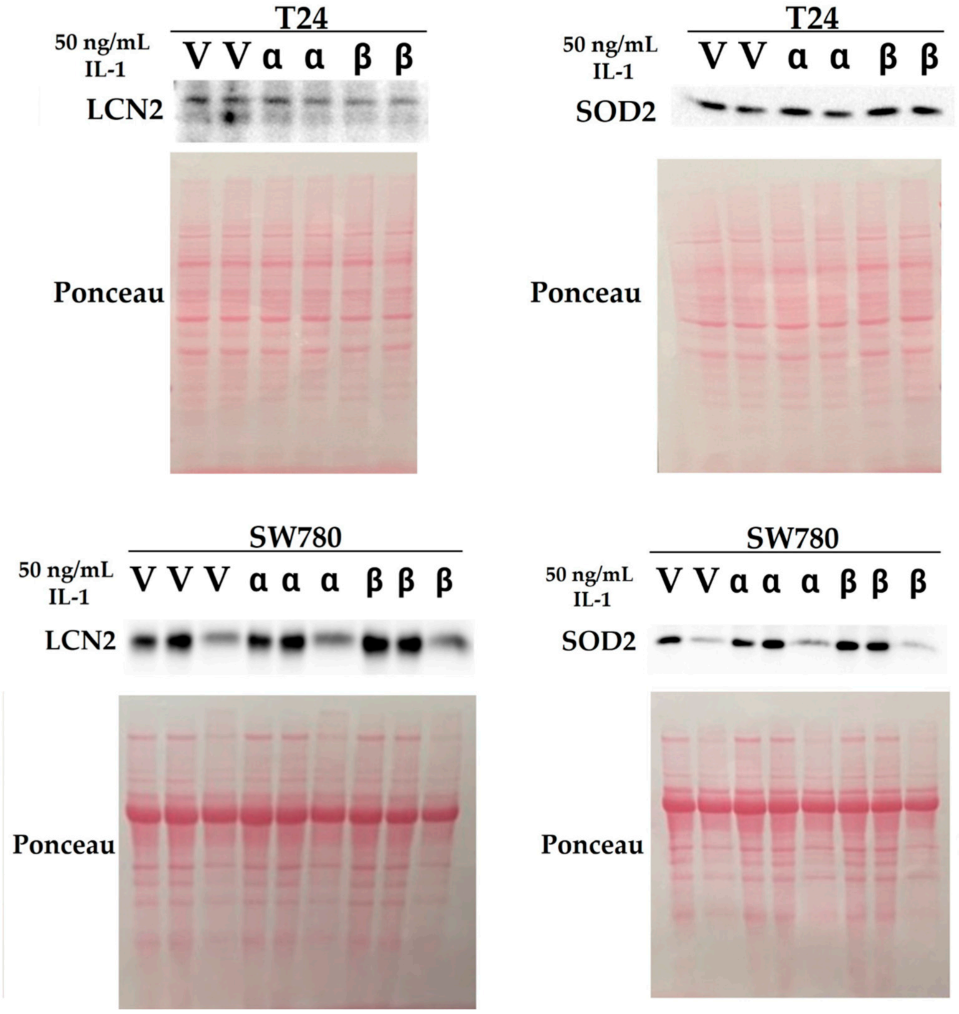

Figure S1. Human BlCa cell lines, T24 and Sw780, are insensitive to exogenous IL-1. T24 and SW780 cells were treated with vehicle control (V) or 50 ng/mL IL-1α or IL-1β for 4 days (2 or 3 biological replicates are shown) and analyzed by western blot for the canonical IL-1-induced genes*, Lipocalin 2 (LCN2)* and *Superoxide Dismutase 2 (SOD2),* which serve as surrogates for IL-1 intracellular activation. IL-1 does induce LCN2 or SOD2 accumulation in T24 or SW780 BlCa cell lines. Ponceau stain is shown as the western blot loading control.

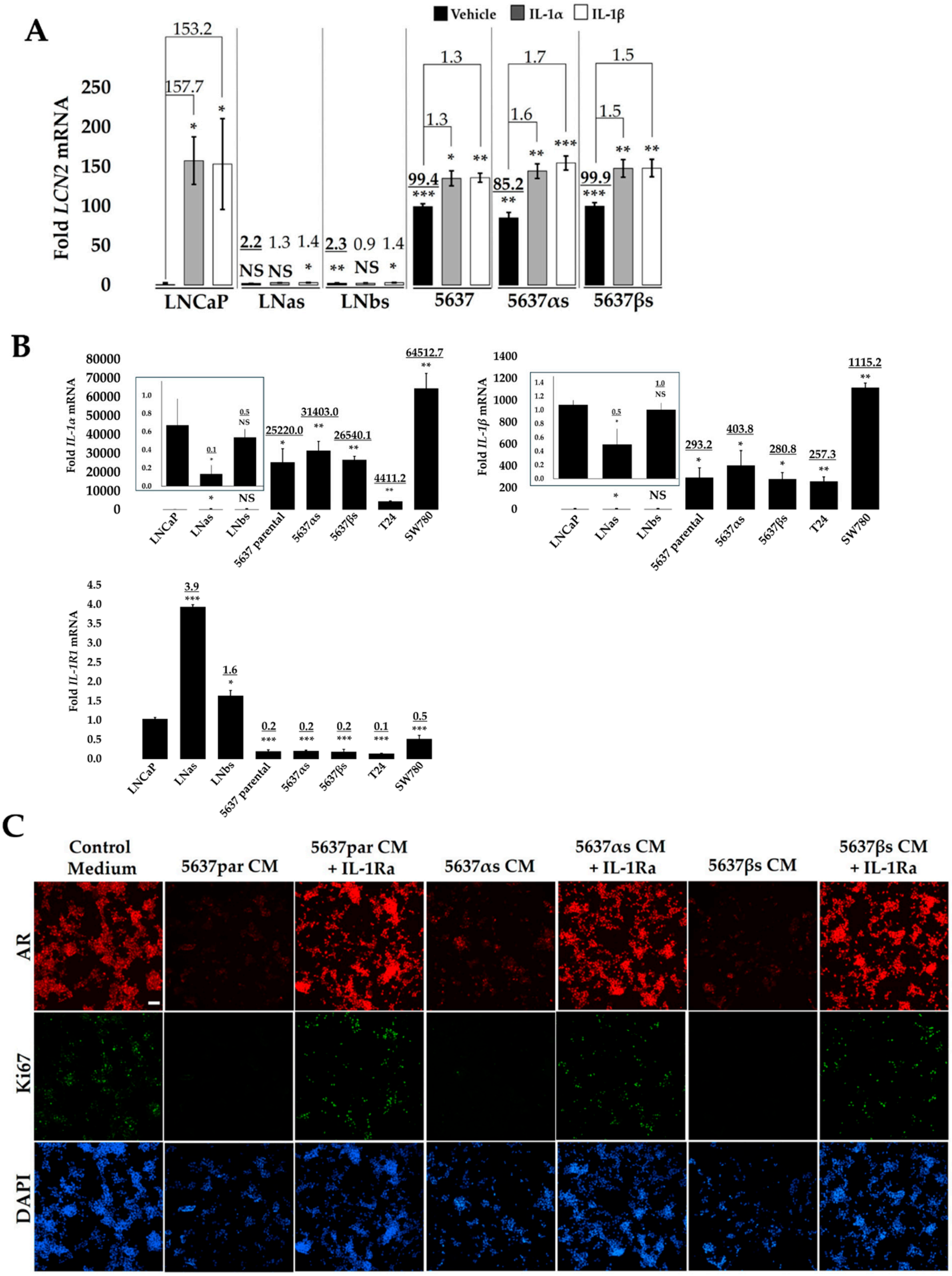

A
Vehicle
IL-1α
IL-1β
Fold LCN2 mRNA
153.2
157.7
2.2
1.3
1.4
2.3
0.9
1.4
NS
**
1.3
1.3
99.4
1.7
1.6
85.2
1.5
1.5
99.9
LNCaP
LNas
LNbs
5637
5637αs
5637βs
B
Fold IL-1α mRNA
25220.0
31403.0
26540.1
4411.2
64512.7
0.1
0.5
Fold IL-1β mRNA
293.2
403.8
280.8
257.3
1115.2
0.5
1.0
Fold IL-1R1 mRNA
3.9
1.6
0.2
0.2
0.2
0.1
0.5
LNCaP
LNas
LNbs
5637 parental
5637αs
5637βs
T24
SW780
C
Control Medium
5637par CM
5637par CM + IL-1Ra
5637αs CM
5637αs CM + IL-1Ra
5637βs CM
5637βs CM + IL-1Ra
AR
Ki67
DAPI

Figure S2. LNCaP cells are more sensitive to acute and chronic IL-1 than 5637 cells. (A) Parental cell lines (LNCaP and 5637) and chronic IL-1 sublines (LNas, LNbs, 5637αs, and 5637βs) were treated acutely with 25 ng/mL (LNCaP, LNas, LNbs) or 75 ng/mL (5637, 5637αs, 5637βs) IL-1α or IL-1β for 3 days. RT-qPCR was performed for canonical IL-1-induced gene, *Lipocalin 2 (LCN2)*, as a surrogate for IL-1 intracellular activation. mRNA fold change is normalized to LNCaP parental vehicle control. Fold change and statistical significance compared to LNCaP parental control (bold) or compared to vehicle control within each cell line is shown. LNCaP parental cells show >150-fold increase in *LCN2* levels in response to IL-1, while 5637, 5637αs, and 5637βs show a 1.3-1.7-fold increase, indicating that LNCaP cells are more sensitive to IL-1. However, basal *LCN2* levels are >85-fold higher in 5637, 5637αs, and 5637βs than LNCaP suggesting that baseline IL-1 signaling is higher in 5637 cells. Finally, as expected, LNas and LNbs, which evolve IL-1 insensitivity due to chronic IL-1 exposure, show substantial attenuated IL-1 response relative to LNCaP parental cells that is not observed for 5637αs or 5637βs in comparison to 5637 parental cells. Thus, chronic IL-1 exposure has a greater effect on the LNCaP cell line background than on the 5637 cell line background. (B) RT-qPCR was performed for basal mRNA levels of *IL-1α, IL-1β,* and *Interleukin-1 Receptor 1 (IL-1R1)* in parental cell lines (LNCaP and 5637) and chronic IL-1 sublines (LNas, LNbs, 5637αs, and 5637βs), in addition to T24 and SW780 BlCa cell lines. mRNA fold change is normalized to LNCaP parental. Insets show LNCaP, LNas, and LNbs. 5637 parental and subline cells have >25,000-fold more IL-1α or >280-fold IL-1β than LNCaP parental and subline cells. Conversely, LNCaP parental and subline cells have >5-fold more IL-1R1 than 5637 parental and subline cells. Thus, the 5637 cell line background is more primed to secrete and facilitate IL-1 paracrine signaling, while the LNCaP cell line background is more primed to respond to IL-1 than the 5637 cell line background. (C) The LNCaP cell line was treated with normal growth medium or conditioned medium (CM) from 5637, 5637αs, or 5637βs cells in the absence or presence of 800 ng/mL IL-1Ra for 2 days. Cells were co-immunostained for AR nuclear accumulation, indicating AR transactivation, and for the proliferation marker, Ki67. 5637 CM, 5637αs CM, and 5637βs CM repress AR and Ki67 nuclear accumulation, which are reversed by the addition of IL-1Ra indicating that 5637, 5637αs, and 5637βs facilitate IL-1 dependent paracrine signaling. RT-qPCR, n = 3 biological replicates; error bars = +/−STDEV; p-value = * ≤0.05, ** ≤0.005, *** ≤0.0005. NS = not significant.
